# A U-Shaped Relationship between Body Mass Index and Dysmenorrhea: A Longitudinal Study

**DOI:** 10.1371/journal.pone.0134187

**Published:** 2015-07-28

**Authors:** Hong Ju, Mark Jones, Gita D. Mishra

**Affiliations:** Centre for Longitudinal and Life Course Research, School of Public Health, the University of Queensland, Brisbane, Queensland, Australia; Medical College of Soochow University, CHINA

## Abstract

**Background:**

Both obesity and dysmenorrhea are prevalent among women. Few population-based longitudinal studies investigate the association between body mass index (BMI) and dysmenorrhea yielding mixed results, especially for obesity. This study aims to investigate the long-term association between BMI and dysmenorrhea.

**Methods:**

9,688 women from a prospective population-based cohort study were followed for 13 years. Data were collected through self-reported questionnaires. The longitudinal association between dysmenorrhea and BMI or BMI change was investigated by logistic regression analysis using generalized estimating equations to account for the repeated measures.

**Results:**

When the women were aged 22 to 27 years, approximately 11% were obese, 7% underweight, and 25% reported dysmenorrhea. Compared to women with a normal weight, significantly higher odds of reporting dysmenorrhea were detected for both women who were underweight (odds ratio (OR) 1.34, 95% confidence interval (CI) 1.15, 1.57) and obese (OR 1.22, 95% CI 1.11, 1.35). Compared to women who remained at normal weight or overweight over time, significant risk was detected for women who: remained underweight or obese (OR 1.33, 95% CI 1.20, 1.48), were underweight despite weight gain (OR 1.33, 95% CI 1.12, 1.58), became underweight (OR 1.28, 95% CI 1.02, 1.61). However the higher risk among obese women disappeared when they lost weight (OR 1.06, 95% CI 0.85, 1.32).

**Conclusions:**

A U-shaped association was revealed between dysmenorrhea and BMI, revealing a higher risk of dysmenorrhea for both underweight and obese women. Maintaining a healthy weight over time may be important for women to have pain-free periods.

## Introduction

Dysmenorrhea is defined as a severe, painful, cramping sensation in the lower abdomen occurring just before or during the menses [1]. It is a common gynaecological complaint, affecting the majority of women of reproductive age with 2–29% having severe pain [2]. As a debilitating condition, it has a major impact on women’s health-related quality of life and social and occupational roles, resulting in significant work and school absences [3, 4]. Considerable economic losses due to dysmenorrhea were estimated resulting from decreased productivity, costs of medications and medical care [5].

Obesity is one of the leading contributors to health loss in Australia [6]. A rapidly increased prevalence was observed over the last decade with 63% of Australian adults being overweight or obese in 2011–2012 [7]. Although obesity has been associated with multiple adverse reproductive health outcomes [8], mixed results have been obtained on its relationship with dysmenorrhea [2]. An earlier systematic review examined the risk factors predisposing women to chronic pain, including 63 studies on dysmenorrhea [9]. It found that BMI < 20 kg/m^2^ was associated with dysmenorrhea, whereas no relationship was demonstrated between BMI > 24 kg/m^2^ and dysmenorrhea. Significant heterogeneity was detected among the studies on the association of dysmenorrhea and BMI > 24 kg/m^2^. A more recent review including mainly community-based cross-sectional studies yielded inconsistent results on the association between BMI and dysmenorrhea, with a large Japanese study revealing a positive association between them and the other smaller studies failed to show any association [2].

To date, only a few population-based longitudinal studies have investigated the association between BMI and dysmenorrhea. Being overweight was found to be a risk factor for the probability of experiencing pain and for increased duration of pain in one study [10], but others failed to show an association between the incidence of dysmenorrhea with BMI [11] or the severity of dysmenorrhea with either weight or height [12].

Given the prevalence of the problems and the mixed findings on their relationship, this study has been undertaken to investigate the longitudinal association between BMI and dysmenorrhea in a large sample of Australian women followed over 13 years.

## Materials and Methods

### Subjects

The study population was from the 1973–78 cohort of the Australian Longitudinal Study on Women’s Health (ALSWH), a prospective cohort study with random sample from the national Medicare database. The 1973–78 cohort included 14,247 women aged 18–23 years at baseline (1996) who were found to be reasonably representative of Australian women of the same age group from the national census despite that women included in the survey were more likely to have tertiary education and less likely to be in the labour force [13]. Questionnaires were sent to participants every three years from Survey 2 onwards, with the most recent survey conducted in 2012. The detailed study methods have been reported elsewhere [14]. ALSWH was approved by the Human Research Ethics Committees of the University of Newcastle and the University of Queensland. Permission to use the data was granted for this study from the Publications, Analyses and Substudies Committee of the ALSWH in August 2012.

As discussed in a previous study [15], dysmenorrhea data from Survey 1 was not included due to evidence of considerable over-reporting of the symptom known as “telescoping” effect common in self-reported data [16]. Therefore data from the last five waves of the survey over 13 years (from Survey 2 in 2000 to Survey 6 in 2012) were included in this study, except that BMI from Survey 1 was used when calculating BMI transition from surveys 1 to 2. Women who were pregnant at surveys 1 to 3 were excluded as the reported weight was not based on their pre-pregnancy weight. Baseline refers to Survey 2 (n = 9,688) hereafter.

### Measurements

All data were collected through self-report at each survey. The outcome of interest, presence of dysmenorrhea, was based on the question: *‘In the last 12 months have you had severe period pain?’* Women were considered to have had a recent history of dysmenorrhea if their response was ‘sometimes’ or ‘often’, and to be symptom-free if their response was ‘never’ or ‘rarely’.

The main exposure of interest was BMI which was calculated as weight in kg divided by the square of height in m. Two measurements in BMI were used to examine its association with dysmenorrhea. First, BMI in kg/m^2^ was categorised as underweight (< 18.5), normal (18.5–24.99), overweight (25–29.99) or obese (≥ 30) based on the recommendations from the World Health Organization [17]. Second, BMI transition was created, based on the change of BMI categories in two successive surveys. As overweight showed no association with dysmenorrhea, it was grouped together with normal BMI in creating BMI transition. Seven BMI transition groups were derived as: 1) remained normal or overweight (reference group); 2) remained underweight or obese; 3) from underweight to normal or overweight; 4) from normal or overweight to underweight; 5) from normal or overweight to obese; 6) from obese to normal or overweight; and 7) varied between normal and overweight.

Other socio-demographic and lifestyle characteristics of the women were collected in each survey and included age, highest level of education, employment, marital status, area of residence, management on income, history of abuse, smoking status, alcohol consumption, illicit drug use and physical activity. The following reproductive characteristics were included: use of oral contraceptives, number of births (live and still), age at menarche and endometriosis. The detailed categorisations of these variables are shown in Table 1.

**Table 1:** Baseline body mass index, other characteristics, and the prevalence of dysmenorrhea among women from the 1973–78 cohort of the Australian Longitudinal Study on Women's Health, aged 22–27 years in 2000.

Characteristics at baseline	Percent^a^	Prevalence of dysmenorrhea (24.5%)	
(N = 9,688)		%^a^	
Body mass index (kg/m^2^) (n = 8,699)			0.001
Underweight (<18.5)	6.7	30.4	
Normal (18.5 to <25)	62.9	24.3	
Overweight (25 to <30)	19.8	26.5	
Obese (≥30)	10.6	28.1	
*Other baseline characteristics*			
Age (years) (n = 9,671)			<0.0001
22–23	37.4	27.3	
24–25	39.9	25.2	
26–27	22.7	21.5	
Highest education (n = 9,325)			0.009
Less than high school	11.3	26.5	
High school / trade certificate	27.2	27.0	
Diploma or higher	61.5	24.0	
Employment status (n = 9,541)			<0.0001
Employed	41.0	22.0	
Unemployed	59.0	27.3	
Marital status (n = 9,623)			<0.0001
Single	53.0	27.4	
Married / De fecto	45.7	22.7	
Separate / divorced / widowed	1.3	20.7	
Area of residence (n = 9,629)			0.05
Urban	55.1	26.1	
Rural	41.1	24.1	
Remote	3.8	22.7	
Language spoken at home (n = 9,576)			<0.0001
English	92.8	24.5	
European	4.7	33.6	
Asian	1.5	27.4	
Other	0.9	40.0	
Coping with income (n = 9,641)			<0.0001
Impossible	3.1	26.4	
Difficult all the time	13.7	33.2	
Difficult sometimes	32.2	26.5	
Not too bad	37.4	22.2	
Easy	13.6	21.7	
History of abuse (n = 9,526)			<0.0001
Yes	39.0	30.8	
No	58.6	21.0	
Don’t want to answer	2.4	30.6	
Smoking status (n = 9,591)			<0.0001
Never-smoker	57.4	23.0	
Ex-smoker	14.5	25.3	
Smoke <10 cigarettes/day	14.5	27.2	
Smoke 10–19 cigarettes/day	8.9	31.6	
Smoke ≥ 20 cigarettes/day	4.7	32.7	
Alcohol consumption (n = 9,606)			0.04
None / rarely	38.0	25.8	
Low-risk drinking	58.2	24.4	
Risky / high-risk drinking	3.8	29.8	
Illicit drug use (n = 9,502)			0.0004
Never	35.8	23.6	
Not in last 12 months	22.0	23.6	
Used in last 12 months	42.2	27.1	
Physical activity (n = 9,421)			0.44
Sedentary	10.5	25.8	
Low	43.8	24.5	
Moderate	23.4	25.2	
High	22.3	26.3	
Oral contraceptive pills (n = 9,532)			<0.0001
Not use	43.9	30.5	
Used	56.1	20.8	
Number of births (n = 9,650)			<0.0001
0	82.2	26.2	
1	10.8	21.8	
≥2	6.9	17.9	
Age at menarche (years) (n = 9,570)			<0.0001
8–11	13.3	30.5	
12–14	74.5	24.4	
≥15	12.2	24.1	
Endometriosis (n = 9,576)			<0.0001
No	96.9	24.0	
Yes	3.1	61.3	

^a^ percent may not add up to 100% due to rounding.

### Statistical analysis

Statistical analyses were performed using SAS version 9.3 for Windows (SAS Institute Inc., Cary, NC). Women’s BMI status at the individual level over time was displayed at each survey using a lasagne plot for longitudinal categorical variables [18]. The prevalence of dysmenorrhea, weighted for area of residence to correct for oversampling of women in rural and remote area, was displayed in a histogram to show its trend over time.

Baseline characteristics of the women in relation to BMI and dysmenorrhea status were compared using χ^2^ test. The longitudinal association between BMI and dysmenorrhea was investigated by logistic regression, taking into account the change of status and the repeated measures of variables over time using generalised estimating equations (GEE). In the BMI transition model, the prevalence of dysmenorrhea at a given survey was modelled in relation to BMI transition between the index survey and the immediately preceding survey. Univariate analysis was performed on all exposure variables, and those which were statistically significantly associated with dysmenorrhea were entered into multivariable-adjusted models. The association of interest was examined after controlling for sociodemographic, lifestyle and reproductive factors, sequentially entered into the models in blocks of variables. Statistical significance was set at p < 0.05.

Due to between 10–32% of women with missing BMI at different time points, sensitivity analysis was performed by comparing the results based on the observed data with results after multiple imputations (MI) for missing data on outcome and covariates. MI procedure in SAS was used as follows: 1) PROC MI (fully conditional specification method) to create a series of 20 imputed data sets; 2) GEE analysis on each of the imputed data set; 3) PROC MIANALYZE to generate a final single set of parameter estimates.

## Results

**Fig 1:**
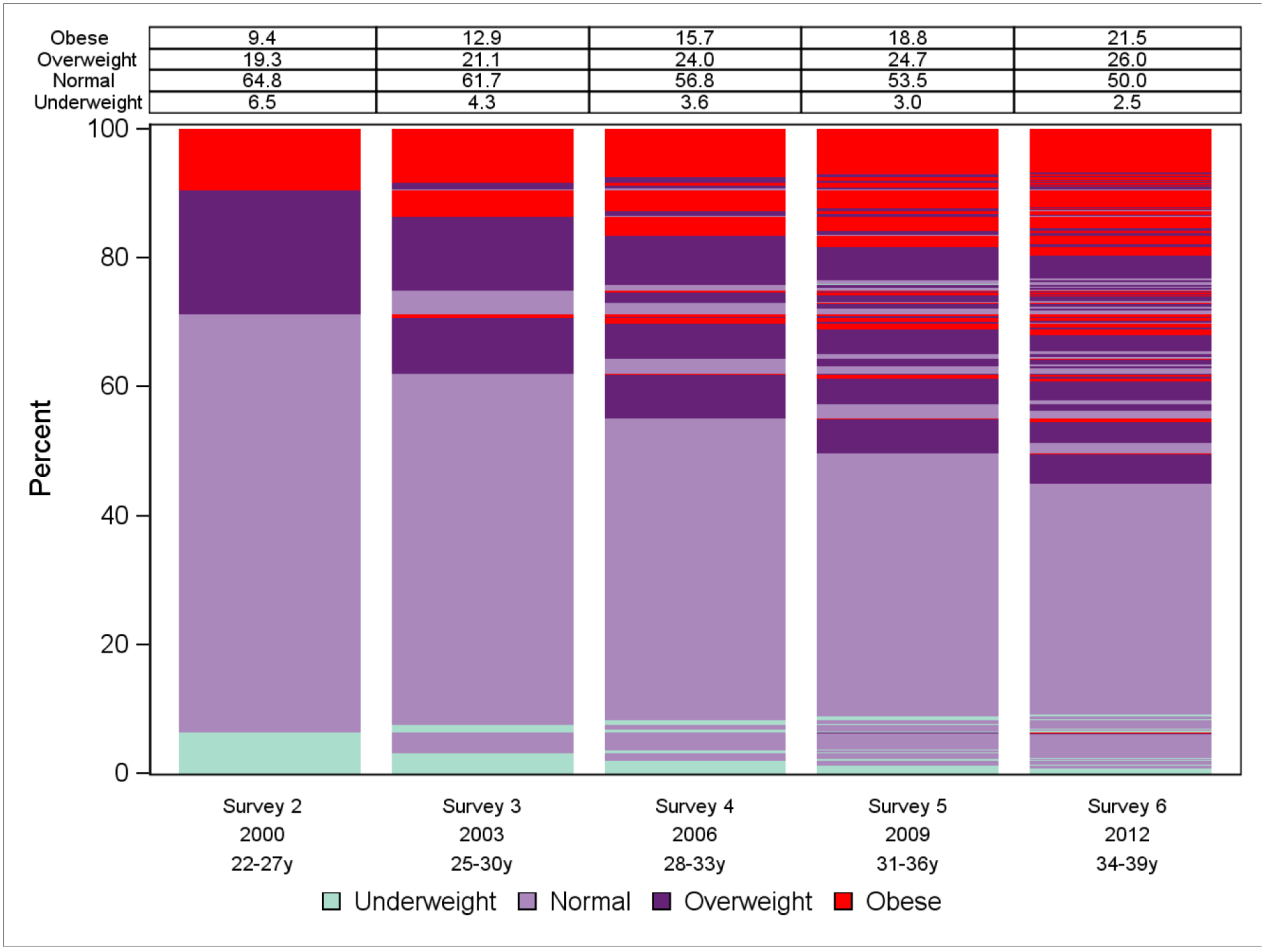
Change of body mass index categories among women from the 1973–78 cohort of the Australian Longitudinal Study on Women's Health from year 2000 (Survey 2) to 2012 (Survey 6). Percentages may not add up to 100% due to rounding.

At baseline, when the women were aged 22 to 27 years, the majority had a BMI in the normal range whereas approximately 20% were overweight and 11% obese (Table 1). A small proportion, 7%, of women was underweight. Over time, an increase in the prevalence of both overweight and obese was observed, especially for obesity (Fig 1). A substantial decrease was detected in the proportion of women classified as normal weight or underweight.

**Fig 2:**
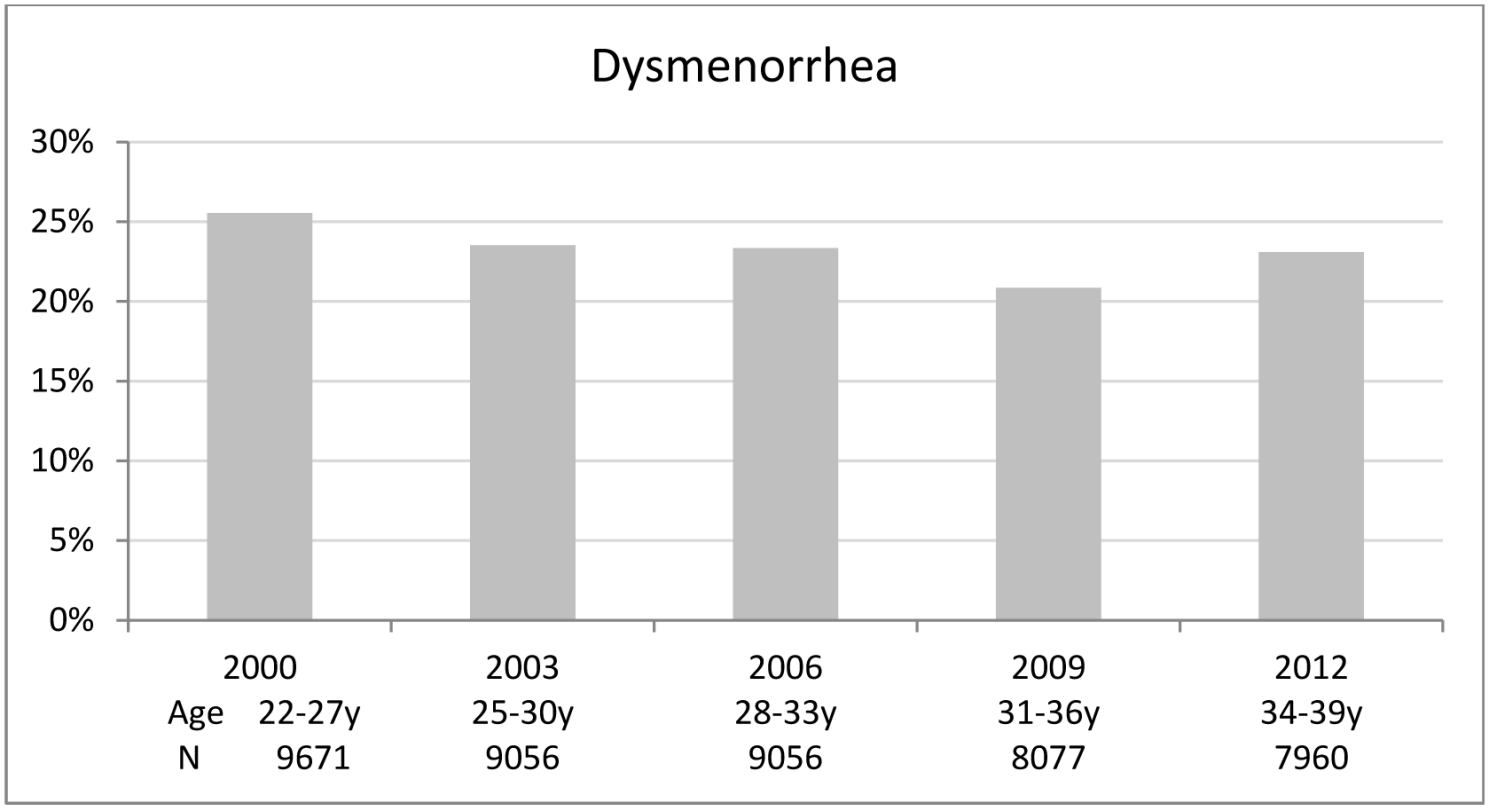
Prevalence of dysmenorrhea among women from the 1973–78 cohort of the Australian Longitudinal Study on Women's Health from year 2000 (Survey 2) to 2012 (Survey 6).

Approximately 25% of the women reported dysmenorrhea when they were aged 22 to 27 years, which remained stable over the study period before a slight increase was observed when they reached 34 to 39 years of age (Fig 2). Baseline characteristics of the women by dysmenorrhea status are presented in Table 1. Women reporting dysmenorrhea were more likely to be underweight or obese, younger, single, employed, heavy smokers, to speak a European language other than English, to have a history of abuse, difficulty managing income, an early age of menarche and to have endometriosis. Women who used OCPs and who had given birth were less likely to report dysmenorrhea.

Over the study period, approximately 77% of women maintained a stable BMI, and 11% changed between healthy (normal or overweight) and risky (underweight or obese) categories (Table 2). The annual average weight change of the women varied according to their BMI transition status. Over time, women who maintained a BMI within normal or overweight group had the most stable weight with an average weight gain of 0.3 kg/year. However a much greater gain of 3.9 kg/year was observed for women whose BMI changed from normal or overweight to obese, and an average weight loss of 3.8 kg/year was seen among women whose BMI changed from obese to overweight or normal.

**Table 2:** Annual average weight change according to body mass index transition groups among the 1973–1978 cohort of the Australian Longitudinal Study on Women’s Health from 1996 to 2012.

	Percent	Annual average weight change (kg)^a^					
Body mass index transition (n = 8,667)		Survey 1–2	Survey 2–3	Survey 3–4	Survey 4–5	Survey 5–6	Overall
Remain normal or overweight	62.7	0.4±1.3	0.5±1.6	0.4±1.6	0.5±1.6	0.3±1.6	0.3±1.2
Remain underweight or obese	14.0	1.2±2.4	1.5±3.0	1.2±3.1	0.8±3.7	0.5±4.0	0.9±2.9
Underweight → normal or overweigh	1.4	0.6±1.4	0.7±1.7	0.5±1.7	0.4±1.7	0.4±1.5	1.7±1.3
Normal or overweight → underweight	5.1	-0.5±1.3	0.1±1.8	0.3±1.8	0.2±1.9	0.1±1.6	-1.5±1.3
Normal or overweight → obese	2.8	1.7±2.4	1.6±3.1	1.3±3.1	1.3±3.1	1.1±3.4	3.9±2.3
Obese → overweigh or normal	1.7	0.8±2.8	0.7±3.6	0.5±3.8	0.4±4.1	-0.5±4.1	-3.8±2.9
Normal ↔ overweight	12.2	0.8±1.8	0.8±2.4	0.7±2.3	0.8±2.3	0.5±2.3	1.1±2.6

^a^ mean±SD

A total of 8,931 women were included in the BMI model. The univariate analysis showed that, compared to those who had a normal BMI, women who were underweight or obese were both at over 40% higher odds to report dysmenorrhea (Table 3). Overweight, however, was not related to dysmenorrhea. After adjusting for potential confounders, the associations were attenuated however significantly higher odds of reporting dysmenorrhea remained for women who were underweight and obese, with 34% and 22% higher odds respectively.

**Table 3:** Univariate and multivariable-adjusted association from GEE analysis between BMI and dysmenorrhea among the 1973–1978 cohort of the Australian Longitudinal Study on Women’s Health from 2000 to 2012.

	Sample (N)	Univariate		Multivariable-adjusted	
BMI		OR	95% CI	OR	95% CI
BMI category (kg/m^2^)	8,931	p < .0001		p < .0001	
Underweight (<18.5)		1.41	1.22, 1.64	1.34	1.15, 1.57
Normal weight (18.5 to <25)		1		1	
Overweight (25 to <30)		1.05	0.98, 1.14	0.99	0.91, 1.07
Obese (≥30)		1.46	1.33, 1.60	1.22	1.11, 1.35
BMI transition	8,579	p < .0001		p < .0001	
Remain normal or overweight		1		1	
Remain underweight or obese		1.54	1.41, 1.72	1.33	1.20, 1.48
Underweight → normal or overweigh		1.36	1.16, 1.60	1.33	1.12, 1.58
Normal or overweight → underweight		1.38	1.10, 1.73	1.28	1.02, 1.61
Normal or overweight → obese		1.21	1.06, 1.37	1.07	0.93, 1.22
Obese → overweigh or normal		1.22	0.99, 1.51	1.06	0.85, 1.32
Normal ↔ overweight		1.08	0.99, 1.19	1.01	0.92, 1.11

BMI, body mass index, GEE, generalised estimating equations.

Multivariable-adjusted analysis estimates the effect of the exposure of interest (body mass index) after controlling for sociodemographics (age, education, employment, marital status, language spoken at home, managing income, and history of abuse), lifestyle factors (smoking, illicit drug use and alcohol consumption), reproductive factors (use of oral contraception, parity, age at menarche, and endometriosis).

**Fig 3:**
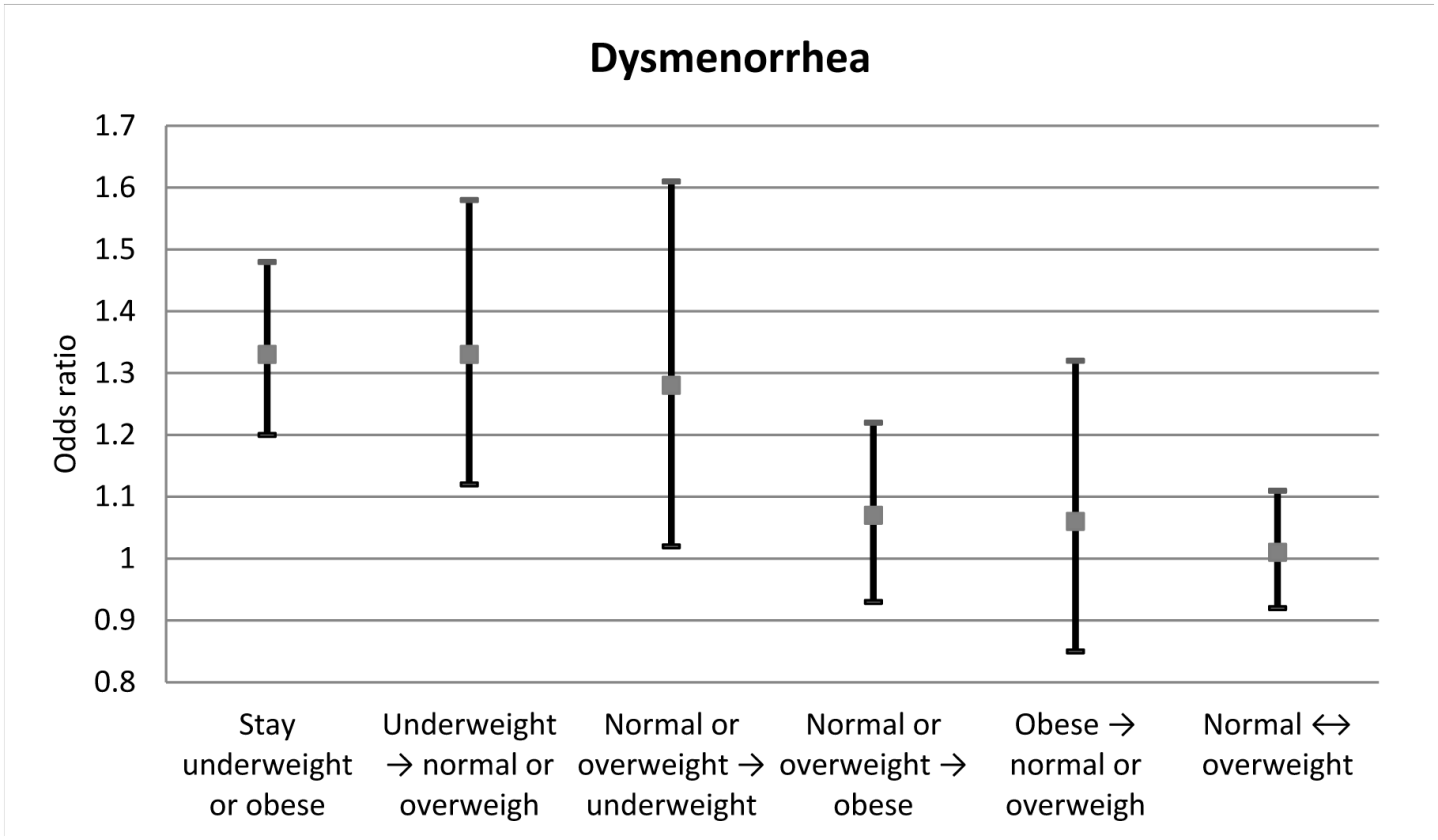
Odds ratio of reporting dysmenorrhea at a survey by BMI transition groups between index surveys and the immediately preceding survey. Reference group = BMI remained as normal or overweight; BMI, body mass index.

Among the 8,579 women included in the BMI transition model, women who remained underweight or obese showed the highest odds of reporting dysmenorrhea compared to women with a BMI staying normal or overweight (Fig 3). Following this, women whose BMI changed from underweight to normal or overweight, and vice versa, also had more than 30% higher odds of reporting dysmenorrhea. These associations were once again attenuated but remained significant in the multivariable-adjusted model. No association was detected for other BMI transition groups.

Results of the sensitivity analysis from the multiple imputed data are presented in S1 Table. Significant association between dysmenorrhea and both underweight and obese remained despite being slightly attenuated, especially for underweight (OR 1.27, 95% CI 1.09, 1.49). For BMI transition, similar results were obtained.

## Discussion

When this group of Australian women were aged 22 to 27 years, every one in 10 was obese. The prevalence of obesity more than doubled to over 23% during the 13 years follow-up, which is consistent with the overall trend in Australia [7]. The prevalence of underweight, however, decreased substantially. Dysmenorrhea affected one-fourth of the women, which remained relatively stable over time. The prevalence is in line with the rate reported for severe dysmenorrhea in a recent review [2].

This study revealed a U-shaped relationship between BMI and dysmenorrhea, with both underweight and obese significantly associated with dysmenorrhea. The result agrees with previous studies on the relationship between low body mass and dysmenorrhea in adolescents and young adult women [19–21]. However previous findings were mixed on the association between dysmenorrhea and overweight or obese, probably due to the varying population included, exposure and outcome measured, and the power of the study [9–12, 22, 23]. Among the few longitudinal studies investigating the relationship, two did not demonstrate any relationship between BMI and either the prevalence or the severity of dysmenorrhea [11, 12]. However, overweight was shown to be a risk factor for dysmenorrhea, doubling the odds of having severe pain or pain lasting longer than 2 days in another study of 165 college women [10]. Our results are in line with this later study since overweight was referred to women with a weight-for-height index above the 90^th^ centile in that study.

We further explored the relationship of BMI transition over time and dysmenorrhea. Compared to women whose BMI remained normal or overweight, women whose BMI remained within the high-risk categories, namely underweight and obese, showed 33% higher risk of dysmenorrhea. The association between dysmenorrhea and obesity disappeared if obese women lost weight, at an average rate of 3.8 kg/year, and moved to a healthier BMI range. However no statistically significant association with dysmenorrhea was shown for women who became obese over the study period. We hypothesised that this may be partly explained by the observation that obese women were more likely to have missing BMI. However it was not confirmed in the sensitivity analysis using multiple imputed data.

On the contrary, women who were previously underweight, despite gaining weight at an average of 1.7 kg/year and moving to a healthier BMI category, still showed 30% higher risk of dysmenorrhea. Similarly, higher risk was shown for normal or overweight women who lost weight and became underweight. The reason for this consistently higher risk of dysmenorrhea for underweight women, despite weight gain, is not apparent. It may be that underweight imposes a stronger and lasting impact on ovarian function at an earlier age or that this group of women may have other underlying conditions that confound or mediate the association. The analyses have adjusted for endometriosis, OCP use, parity and history of abuse (including sexual, emotional and physical). We further examined the relationship between BMI and a number of other potential underlying conditions and found that although they were more prevalent among underweight women compared to normal weight women, they were not different among women who were underweight or obese. Nevertheless, contrasting the radical changes in the perception of body size which have pressured women to be super slim [22], this study shows that very thin women may suffer from distressing period pain repeatedly over their reproductive life. There is sparse literature on the association of dysmenorrhea and BMI transition, which may be an area for future study.

There is complex interaction between body fat and steroid hormones, thus the endocrine control of menstruation [24]. As a result, the underlying mechanisms of the association between BMI and dysmenorrhea are not well understood, and they may differ in underweight and obese women. Nevertheless, a certain amount of body fat appears to be important to maintain normal ovulatory cycles with both too much and too little fat being associated with the disruption of their reproductive health [25, 26]. There are several known mechanisms on the influence of adipose tissue on ovulation and menstrual cycle: 1) adipose tissue converts androgens to oestrogen by aromatisation; 2) body weight influences the direction of oestrogen metabolism with very thin women making less potent and obese women more potent forms of oestrogen; 3) obese women have a diminished capacity for oestrogen to bind to sex-hormone binding globulin (SHBG) which inactivates oestrogen, resulting in an elevated percentage of free serum oestradiol [27].

The primary disease pathogenesis for dysmenorrhea has been related to increased prostaglandins in the menstruating uterus, leading to reduced endometrial blood flow and subsequent pain [4]. There is suggestion that endometrial thickness may be influenced by adiposity through its oestrogen-mediated effect [8, 10]. Body weight has been inversely correlated with serum SHBG concentrations [28], and diminished SHBG or elevated serum oestrogen potentially increases oestrogenic stimulation of the endometrium, prompting proliferation of tissues that produce prostaglandins, particularly PGF_2α_ [29]. However an inverse relationship was also shown between BMI and total oestrogen [30], supporting the theory that oestrogen/progesterone ratio, instead of oestrogen alone, may underlie the pathogenesis of dysmenorrhea [31]. Alternatively, adipose tissue produces adipokines, the signalling molecules which vary in their production with adipose mass and may directly cause impaired ovarian function through altering the hypothalamic-pituitary-ovarian axis signalling, resulting in disrupted menstruation [8]. There is evidence that menstrual irregularity is higher in both girls with low and high BMI [22, 27], and having menstrual irregularity has been associated with dysmenorrhea [9].

Furthermore, pain ‘catastrophising’, the role of central nervous system contributions to increased menstrual pain intensity, was also suggested as potential cause for dysmenorrhea [4]. Under this theory, there may be psychological stress related to being underweight or obese which may cause differences in pain perception and sensitivity between these women and normal weight women, resulting in different subjective experience of pain [24, 27]. In addition, there is evidence that ovarian hormones (especially oestrogens) play a role in modulating a range of chronic pain conditions through affecting concentration of oestrogen receptors in the spinal cord or the corresponding brain regions, or through interacting with different neurotransmitters that modulate pain perception [32]. Generally a low oestrogen milieu is suggested to exacerbate the severity of chronic pain [32], which may be particularly relevant in the association between underweight and dysmenorrhea.

### Strengths and limitations

This is the first large population-based longitudinal study to investigate the association between BMI and the risk of dysmenorrhea, revealing higher risk for both extreme ends of BMI. It further explored the association of BMI transitions over time and dysmenorrhea. The representative sample makes the results generalisable to women with similar characteristics. Nevertheless the study has a number of limitations. Firstly, all the data are self-reported based on questionnaires, which may be subject to reporting bias. However self-reported dysmenorrhea has been correlated well with prospectively recorded data [33], and our estimated prevalence of dysmenorrhea is in line with that reported in the literature [2], providing justification on the validity of the data. In addition, given the longitudinal nature and large size of the study, self-report may be the most feasible means of data collection. Secondly, data over the past 12 months were collected retrospectively at each survey wave, which may subject the data to recall bias. However, daily symptom reporting over an extended evaluation period may not be practical in this large population setting. The long period of observation and repeated measures may also offset the disadvantage of the absence of daily rating. Thirdly, data on other measures of adiposity (eg., waist-to-hip ratio, abdominal circumference) were not routinely collected in the survey, rendering our ability to stratify our analysis according to markers of visceral adiposity and explore their relationship with dysmenorrhea. Fourthly, given that surveys were conducted every 3 years, there is possibility that some women may have transited among BMI categories in-between surveys. Fifthly, a high attrition (32%) at Survey 2 may have introduced potential selection bias. Comparison of the prevalence of dysmenorrhea at Survey 1 among respondents (44.6%) and non-respondents (42.8%) showed no evidence of differential drop-out, indicating the random nature of attrition for the outcome of interest.

In conclusion, this longitudinal study demonstrates a U-shaped association between dysmenorrhea and BMI, revealing a higher risk of dysmenorrhea for both underweight and obese women. Further, women who remained underweight or obese over time maintained the higher risk, whereas the risk disappeared when obese women lost weight and acquired a healthier BMI. On the other hand, women with a healthier BMI but became underweight appeared to acquire a higher risk of dysmenorrhea. However future research is needed to replicate the findings. From a public health perspective, obesity certainly contributes to a greater burden of disease than underweight, given the larger proportion of women affected. Maintaining a healthy weight over time may be important for women to have pain-free periods, and thus an improved reproductive health.

## Supporting Information

S1 Table Results from multiple imputations on the association between BMI and dysmenorrhea, from GEE analysis, among the 1973–1978 cohort of the Australian Longitudinal Study on Women’s Health from 2000 to 2012. BMI, body mass index; GEE, generalised estimating equations.
(DOCX)
