# Supplementary material for: A U-Shaped Relationship between Body Mass Index and Dysmenorrhea: A Longitudinal Study
Source: PLoS One. 2015 Jul 28;10(7):e0134187. doi: 10.1371/journal.pone.0134187 (PMC4517870; doi:10.1371/journal.pone.0134187)
Supplement: S1 Table — BMI, body mass index; GEE, generalised estimating equations. (DOCX) [file pone.0134187.s001.docx]

A U-shaped relationship between body mass index and dysmenorrhea: a longitudinal study

S1 Table: Results from multiple imputation on the association between BMI and dysmenorrhea, from GEE analysis, among the 1973-1978 cohort of the Australian Longitudinal Study on Women’s Health from 2000 to 2012

| **BMI** | **N** | **OR** | **95% CI** |
| --- | --- | --- | --- |
| BMI category (kg/m^2^) | 9,688 |  | |
| Underweight (<18.5) |  | 1.27 | 1.09, 1.49 |
| Normal weight (18.5 to <25) |  | 1 |  |
| Overweight (25 to <30) |  | 1.03 | 0.96, 1.10 |
| Obese (≥30) |  | 1.20 | 1.10, 1.31 |
| BMI transition | 9,021 |  | |
| Stable (normal or overweight) |  | 1 |  |
| Stay underweight or obese |  | 1.33 | 1.20, 1.47 |
| Underweight → normal or overweigh |  | 1.33 | 1.13, 1.55 |
| Normal or overweight → underweight |  | 1.27 | 1.03, 1.58 |
| Normal or overweight → obese |  | 1.08 | 0.95, 1.23 |
| Obese → overweigh or normal |  | 1.09 | 0.89, 1.34 |
| Normal ↔ overweight |  | 1.04 | 0.95, 1.14 |

BMI, body mass index; GEE, generalized estimating equations.

The estimates on the association of dysmenorrhea and the exposure of interest (BMI and BMI transition) were adjusted for sociodemographics (age, education, employment, marital status, managing income, and language spoken at home), lifestyle factors (smoking, illicit drug use and alcohol consumption), reproductive factors (use of oral contraception, parity, age at menarche, endometriosis, and history of abuse).
